# Statins and Cancer: A Complex Relationship Worth Exploring

**DOI:** 10.3390/ph16111570

**Published:** 2023-11-07

**Authors:** Jacek Bil

**Affiliations:** Department of Invasive Cardiology, Centre of Postgraduate Medical Education, 02-507 Warsaw, Poland; biljacek@gmail.com; Tel.: +48-47-722-11-00

This Special Issue, entitled “Statins and Cancer”, aims to demonstrate recent and new advances and future trends in using statins in the field of oncology.

Statins, a class of drugs primarily prescribed to lower cholesterol levels and reduce the risk of heart disease, have been the subject of extensive research over the years. The first statin, lovastatin, was introduced to therapy in 1987, and statins quickly became one of the most commonly prescribed classes of drugs worldwide. Currently, six forms of statin are available on worldwide markets: atorvastatin, rosuvastatin, pravastatin, pitavastatin, simvastatin, and fluvastatin. After the publication of prospective epidemiological studies, some concerns arose about the association of statins with an increase in noncardiac mortality, especially cancer-related mortality [1]. In addition, some in vitro studies suggested that statins might interfere with anticancer drugs, hampering their effectiveness, especially in the case of new targeted therapies like monoclonal antibodies [2,3]. However, the large ‘’Heart Protection Study’’ results were published, in which investigators found no association between cancer incidence and the administration of statins [4,5].

Nowadays, statins efficacy in managing cardiovascular health is well-established, and a growing body of evidence also suggests that statins may have a role to play in cancer prevention and treatment [5,6,7]. The relationship between statins and cancer is complex and multifaceted, making it an area of ongoing scientific investigation, as shown in this Special Issue.

One of the most intriguing aspects of the statin-cancer connection is the potential for these drugs to inhibit the growth and progression of various cancer types. Statins are thought to exert their anti-cancer effects through several mechanisms, including reducing inflammation, inhibiting cell proliferation, and influencing cellular processes that are crucial for cancer development [8]. Some studies have indicated that statins might be particularly beneficial in preventing certain types of cancer, such as breast, prostate, and colon cancer [9,10,11,12,13]. Some other studies also showed that statin use is beneficial even in advanced stages of cancer [14].

However, the evidence on statins and cancer is far from conclusive, and many questions remain unanswered. Some research suggests that the protective effect of statins against cancer may be dose-dependent, with higher doses showing stronger associations. Furthermore, the exact mechanisms by which statins influence cancer biology are still debated, and the optimal timing and duration of statin use for cancer prevention are unclear [15,16].

Acknowledging that not all studies have consistently supported the notion that statins are unequivocally beneficial for cancer prevention or treatment is essential. Some investigations have yielded mixed results, with certain cancers showing no significant reduction in risk associated with statin use. Additionally, concerns have been raised about the potential side effects of long-term statin use, underscoring the need for a careful risk-benefit analysis, particularly in individuals without cardiovascular risk factors [6,17,18].

Ongoing research efforts are essential to better understanding the relationship between statins and cancer. Large-scale clinical trials encompassing diverse populations and various cancer types are needed to provide more robust evidence. Furthermore, research should focus on identifying specific patient subgroups that might derive the most significant benefit from statin therapy in the context of cancer prevention and treatment [19].

This Special Issue highlighted that the relationship between statins and cancer is an intricate and evolving field of study [5,6,11,12,15,16,17,18,19]. While the potential benefits of statins in preventing and treating cancer are promising, they are not yet well established across the board. Patients and healthcare providers should weigh the known cardiovascular benefits of statins against the uncertain advantages in cancer prevention and treatment. As research continues, a more comprehensive understanding of the role of statins in cancer will undoubtedly emerge, potentially opening up new avenues for cancer management. Until then, the cautious use of statins in the context of cancer should be guided by the available evidence and individual patient considerations.
